# Impulsivity in adolescent girls diagnosed with trichotillomania: an evaluation of clinical and neuropsychological characteristics

**DOI:** 10.1007/s00787-023-02354-x

**Published:** 2024-01-09

**Authors:** Hande Günal Okumuş, Devrim Akdemir, Rahime Duygu Temeltürk, Makbule Esen Öksüzoğlu

**Affiliations:** 1 Hacettepe University, Ankara, Turkey; 2 Ankara University, Ankara, Turkey; 3 Kastamonu Training and Research Hospital, Kastamonu, Turkey

**Keywords:** Trichotillomania, Impulsivity, Motor inhibition, Decision-making, Neuropsychological assessment, Adolescents

## Abstract

The aim of this study is to compare the clinical and neuropsychological features of impulsivity in adolescent girls with trichotillomania (TTM) and healthy controls, and to assess the relationships between the severity of TTM and the impulsivity/concomitant symptoms of anxiety and depression. The study sample consisted of 43 adolescent girls who were 12 to 18 years old. The Kiddie-Schedule for Affective Disorders and Schizophrenia for School Age Children-Present and Lifetime Version DSM-5 (K-SADS-PL) was administered to the adolescents and their parents. All of the participants completed a sociodemographic data form, the Revised Children’s Anxiety and Depression Scale-Child Version (RCADS-CV), the Barratt Impulsiveness Scale-Brief (BIS-Brief), and the Massachusetts General Hospital Hairpulling Scale (MGH-HPS). The Eriksen Flanker task, the Stop Signal Reaction Time (SSRT) task, the Go/No-Go task, and the Balloon Analog Risk Task (BART) were used to assess behavioral impulsivity. The adolescents with TTM reported higher levels of impulsivity and anxiety/depression symptoms than the healthy controls, and they also performed worse on the behavioral tasks. While there were no relationships between clinical and behavioral impulsivity and TTM severity, social anxiety symptoms were the most important predictor of the severity of TTM. It seems important to better understand the role of impulsivity in the onset and persistence of TTM symptoms in adolescents.

**Supplementary Information:**

The online version contains supplementary material available at 10.1007/s00787-023-02354-x.

## Introduction

Trichotillomania (TTM), or hair-pulling disorder, is a psychiatric disorder characterized by repetitive hair-pulling behavior that leads to hair loss and/or thinning and unsuccessful attempts to reduce/stop hair-pulling and is closely associated with functional impairment [2]. TTM was introduced into the psychiatric classification system under the rubric of “Impulse Control Disorder Not Elsewhere Classified” in DSM-III. In the most recent revision, DSM-5, TTM is categorized in the taxonomy of Obsessive Compulsive and Related Disorders (OCRDs). However, the ICD-11 Working Group has proposed including TTM and skin-picking disorder (SPD) in a new subgroup called “body-focused repetitive behavior disorders (BFRBs)” [38]. A recent meta-analysis comprehensively evaluated 30 studies of adolescents and adults and found that the prevalence of any kind of hair-pulling behavior was 8.8%, while the prevalence of TTM specifically was 1.1% [72]. A review of the medical literature reveals a limited number of studies on the epidemiology, etiology, clinical course, and treatment of TTM, despite the fact that it appears to be a long-standing psychiatric disorder, particularly among children and adolescents.

High rates of comorbidity with the psychiatric disorders that are closely related to impairment in impulse control (e.g., attention deficit hyperactivity disorder, or ADHD) [12, 62], high rates of alcohol and substance use disorders in the first-degree relatives of individuals diagnosed with TTM or themselves [34, 61], reports of relief and pleasure during hair-pulling episodes in some individuals, and successful treatment with dopamine receptor blockers indicate that TTM is strongly associated with impulsivity [27, 39]. Impulsivity is generally characterized by a tendency to act without considering potential negative consequences and includes inappropriate, ill-considered, hasty actions leading to undesirable outcomes [23]. The construct of impulsivity has five discrete cognitive domains: *motor impulsivity* (involving challenges in inhibiting motor responses), choice impulsivity (the postponement of a larger prospective reward in favor of an immediate modest reward), disadvantageous decision*-*making (a hesitancy in assuming appropriate risks due to challenges in evaluating options), interference control (difficulties with suppressing distracting stimuli), and *reflex impulsivity* (inadequate information gathering before decision-making) [25]. Studies investigating impulsivity and related neurocognitive mechanisms in TTM have primarily focused on the concept of impairment in motor response inhibition [51, 56]. Numerous studies using behavioral tasks in adults with TTM have consistently demonstrated impairments in motor response inhibition [9, 11, 35, 56]. Similarly, neuroimaging studies in TTM have revealed structural abnormalities in the brain regions involved in motor response inhibition. Excess cortical thickness in the right inferior frontal gyrus has been found in adults with TTM, and it has been emphasized that structural changes in this region play a central role in the pathophysiology of TTM [56]. Another study found a statistically significant decrease in bilateral cerebellar volume in adults with TTM, and smaller cerebellar volume has been associated with greater symptom severity [46]. Volumetric differences have also been reported in the putamen and amygdala [43]. The supplementary motor area (SMA) also plays a critical role in motor planning and response inhibition. Dysfunction in the SMA can lead to the disappearance of inhibition in the striatum, resulting in symptoms similar to those of obsessive–compulsive disorder (OCD) [13]. Consistent with this finding, it has been reported that application of the repetitive transcranial magnetic stimulation (rTMS) to the SMA regions in an adult with TTM resulted in significantly reduced hair-pulling symptoms [3]. Given these findings in adult populations, the literature often emphasizes the importance of the association between impaired motor response inhibition and TTM and advocates the need to assess motor response inhibition carefully when developing therapeutic interventions [9, 33, 68]. However, there are limited numbers of behavioral studies assessing motor response inhibition in children and adolescents with TTM, and their findings are conflicting [6, 49, 78].

Given the multidimensional nature of impulsivity, assessing components beyond motor response inhibition, particularly the interference control and decision-making processes, will contribute to a more comprehensive understanding of the neurocognitive facets of the disorder and promote the development of effective and tailored therapeutic modalities for TTM. Studies evaluating the decision-making processes of patients with TTM are extremely limited, and the data they report are contradictory [10, 17, 48]. Studies assessing interference control using the Stroop and Eriksen flanker tests have been conducted primarily with populations of with OCD and major depressive disorder (MDD) [18, 42, 55, 58]. To the best of our knowledge, there are no studies that investigate the interference control and/or decision-making processes of children and adolescents with TTM. Therefore, the primary goal of this study is to compare the clinical and neuropsychological characteristics (motor impulsivity, interference control, and disadvantageous decision-making) of impulsivity in adolescent girls with TTM and healthy controls. Its secondary goal is to assess the relationships between the severity of TTM and the impulsivity/concomitant symptoms of anxiety and depression.

Here are this study’s hypotheses: (1) adolescents with TTM will exhibit higher levels of impulsivity, assessed via both self-report scales and behavioral tasks; (2) adolescents with TTM will perform worse on behavioral tasks, particularly those that assess motor response inhibition and disadvantageous decision-making; (3) adolescents with TTM will have more comorbid anxiety and depressive symptoms than healthy controls, symptoms which will be correlated to the symptom severity of their TTM, and (4) clinical and behavioral impulsivity will be the best predictor of the symptom severity of TTM.

## Methods

### Participants

The research group was recruited from 12 to 18 years old adolescents who visited the Child and Adolescent Psychiatry outpatient clinic of University from 2021 to 2023. The inclusion criteria for the TTM group were; having a current diagnosis of TTM according to DSM-5, being in the acute phase, applying to our clinic for treatment, and being 12–18 years old. The exclusion criteria for the TTM group were; having a diagnosis of any neurological or chronic medical condition or other psychiatric disorder (psychotic disorders, bipolar disorder, autism spectrum disorders, OCD, ADHD, alcohol/substance-related disorders, stereotypic movement disorders, and tic disorders), having a history of head trauma, receptive and expressive language problems, clinically abnormal mental development, and current use of stimulants, atomoxetine, and/or antipsychotics. Patients with MDD and anxiety disorders were not excluded because of their high comorbidity rates with TTM [19]. There were only three adolescent boys with TTM. To mitigate the potential influence of gender on impulsivity, they were excluded from the initial cohort. Furthermore, three patients with a comorbid diagnosis of ADHD and one patient who declined to participate and had hyperthyroidism, which can affect cognitive performance, were subsequently excluded from the study. Ultimately, 7 of the 30 patients with TTM were excluded from the study, and the research group consisted of 23 adolescent girls with TTM.

The healthy control group consisted of age-matched adolescent girls who came to the department for counseling and voluntarily agreed to participate in the study. The healthy group members did not meet the diagnostic criteria for any psychiatric diagnosis as assessed during semi-structured clinical psychiatric interviews. Five adolescents who were going to be included in the control group were excluded from the study, because they were diagnosed with psychiatric disorders [one with ADHD, one with ADHD and speech fluency disorder, one with generalized anxiety disorder (GAD), one with MDD, and one with Social Anxiety Disorder (SAD)]. The control group included 20 healthy adolescent girls.

The ethical committee of University approved the protocol (June 21, 2022/No: GO 22/481), and written informed consent was obtained from all the adolescents and their parents.

### Measures

A sociodemographic and clinical data form was designed by the researchers to collect sociodemographic characteristics such as the age and psychiatric history of the adolescents and their parents, and the parents’ educational and occupational status. In addition, the form was used to collect clinical information relevant to TTM, including the duration of symptoms, time of diagnosis, use of pharmacological agents, specific hair-pulling sites, and the presence of trichophagia.

The Kiddie-Schedule for Affective Disorders and Schizophrenia for School Age Children-Present and Lifetime Version DSM–5, K-SADS–PL-Turkish is a semi-structured interview form designed to detect current and lifetime psychopathologies in children and adolescents [45] and adapted according to DSM–5 for the Turkish sample [74]. The K-SADS-PL was administered to the adolescents and their parents by a child and adolescent psychiatrist to identify comorbid psychiatric disorders in the research group and to exclude the adolescents with psychiatric disorders in the healthy control group.

The Revised Children’s Anxiety and Depression Scale-Child Version (RCADS-CV) is a self-report questionnaire developed to measure anxiety and depression symptoms among children and adolescents. The RCADS-CV consists of 47 items in six subscales: separation anxiety disorder (SPD, nine items), SAD (seven items), GAD (six items), Panic Disorder (PD, nine items), OCD (six items), and MDD (ten items). The RCADS-CV is a valid and reliable psychometric tool for Turkish samples aged 8–17 years [31].

The Massachusetts General Hospital Hairpulling Scale (MGH-HPS*)* is a 7-item self-report questionnaire widely used to measure the severity of symptoms and treatment response in TTM [47]. The scale, which assesses the past week, has a single-dimensional structure. Each item is rated on a scale from 0 to 4, with higher scores indicating greater severity. The reliability and validity of the Turkish version of the MGH-HPS have been demonstrated [59].

The Barratt Impulsiveness Scale-Brief (BIS-Brief), which was developed by Steinberg and his colleagues, is a short version of the BIS-11 [70]. Since the BIS-11 has specific items that are not suitable for adolescents, the BIS-Brief is a preferable alternative for measuring impulsivity in this population. In particular, removing the inappropriate items makes all eight components of the BIS-Brief suitable for adolescents. This measure serves as a valuable tool for assessing impulsivity in both clinical and non-clinical adolescent cohorts. The 8-item BIS-Brief is a self-report questionnaire with two separate subscales for poor self-regulation and for impulsive behavior. Responses are scaled from 1 to 4 and include four reverse-coded items. The BIS-Brief is a valid and reliable psychometric tool for Turkish adolescents [4].

E-Prime is a software program designed for collecting and analyzing data systematically with increased reliability and standardization. Its notable features include an intuitive user interface, including Turkish, temporal precision that ensures fidelity in data collection and ease of data export [63]. The current version, E-Prime 3.0, was used in the present study, and four tasks from the battery were administered to all participants to assess different dimensions of impulsivity.

The Eriksen flanker task is a behavioral task designed to measure interference control [22]. In this task, the target stimulus is centrally located and flanked by three types of non-target stimuli. The first mode is the congruent stimulus mode, in which the direction of the non-target stimulus is the same as that of the target stimulus. In the incongruent stimulus mode, the direction of the non-target stimulus is opposite to that of the target stimulus. Thus, it is harder to choose the correct response in the incongruent mode than it is in the congruent mode. The difference in performance between congruent and incongruent stimulus conditions is called the flanker effect, and the magnitude of the flanker effect is associated with response inhibition. Finally, the neutral stimulus does not require the same response as the target stimulus, nor does it elicit a response conflict [55]. Data collection includes the determination of the percentage of congruent stimulus accuracy, the percentage of incongruent stimulus accuracy, and the flanker effect.

The Stop Signal Reaction Time (SSRT) task is a widely used behavioral task for measuring motor response inhibition, which quantifies the participants’ ability to specifically suppress already initiated motor responses. In this task, participants are instructed to respond to a go signal. However, they must stop this response when they hear a stop signal tone indicated by an auditory beep. SSRT is the primary outcome measure, and a longer SSRT is associated with less motor response inhibition [76]. Data collection includes the determination of the median correct reaction time for successful go trials, the percentage of correct go responses, the percentage of effectively inhibited stop responses, and SSRT.

The Go/No-Go task is a behavioral task designed to measure motor response inhibition. This task consists of a series of trials in which participants are instructed to click the circle once when they hear two beeps and to click the circle twice when they hear one beep. The instructions are then modified by asking participants to double-click the circle when they hear one beep and to do nothing when they hear two beeps [79]. The dependent variables are the percentage of go accuracy, correct reaction time for successful go trials, and percentage of no-go accuracy.

The Balloon Analog Risk Task (BART) is often used with adolescents to assess decision-making in conditions of uncertainty. At the start of the task, a balloon appears on the screen, and the participant is asked to press the button to inflate the balloon. Each click inflates the balloon by one unit, and the participant scores points while it inflates. However, there is a risk that the balloons may burst at any time, and if the balloon bursts before the participant collects the points scored, all the points are forfeited. High scores on all the variables used in the BART are associated with greater risk-taking behavior [50]. The data are evaluated by determining the maximum numbers of pumps, the numbers of adjusted pumps, the numbers of exploded balloons, and total scores.

### Data analyses

This study’s power analysis was performed by evaluating the neurocognitive performance of adults with TTM on the SSRT task in the literature [11, 35, 57]. There are studies with power results ranging from 0.29 to 1.58, so with an average power of 0.72 and an acceptable alpha value of 0.05, the required number of samples for the SSRT task was determined to be 31. This study also conducted a power analysis by evaluating the neurocognitive performance of adults with TTM on the BART. No sample studies with significant results for patients with TTM were found in the literature, but given its close relationship with impulse control disorder, a study conducted with alcohol use disorder patients [7] found that a required sample size of 21 for the BART was obtained when the power was 0.80 with an acceptable alpha value of 0.05. Since our study’s sample included 43 participants, the required power was met for both behavioral tasks.

The Statistical Package for the Social Sciences (SPSS) version 26.0 was used for the statistical analysis of the data. The normality of the data distribution was assessed using either Kolmogorov–Smirnov or Shapiro–Wilk statistics. Means and standard deviations were used for variables with normal distribution in descriptive analyses. Medians, interquartile ranges, and minima and maxima were used for variables with non-normal distributions, and numbers and percentages were used for qualitative variables. The* t* test was used when parametric test assumptions were met, and the Mann–Whitney *U* test was used when parametric test assumptions were not met. Either the Chi-square test or Fisher’s exact test were used to compare the two groups’ data. ANCOVA was used to compare the groups’ neurocognitive test scores, while their total depression and anxiety scores were used as covariates. Pearson’s and Spearman’s correlation tests were used to examine the relationships between the two groups’ measurement values. To examine the variables that were found to be statistically significant in the correlation analyses of TTM severity, more than one simple linear regression analysis was performed, and the variables that were predictive of TTM severity were identified.

## Results

The sociodemographic characteristics of the TTM and healthy control groups are shown in Table 1. Comparison of the groups’ family histories of psychiatric disorders found a statistically significant difference between them (Table 1): six relatives of the TTM group were diagnosed with TTM. The distribution of the groups’ psychiatric diagnoses is shown in Table S1 (Supplementary Table S1).

**Table 1:** The groups’ sociodemographic characteristics

Sociodemographic variables	TTM (*n* = 23) Median (IQR)/*n* (%)	Control (*n* = 20) Median (IQR)/*n* (%)	*U*/*χ*^2^	*p*
Child’s age (years)^a^	15.5 (14–17)	15.5 (14.1–16.9)	217.50	0.766
Mother’s age (years)^a^	39 (34–44)	44 (38.8–49.1)	178.50	0.213
Father’s age (years)^a^	44 (40–48)	46 (43.1–48.9)	174.50	0.179
Socioeconomic status^b^, *n* (%)				
Low	2 (8.7)	1 (5)	0.35	1
Medium	9 (39.1)	8 (40)		
High	12 (52.2)	11 (55)		
Family history of psychiatric disorders^c^, *n* (%)				
Absent	11 (47.8)	16 (80.0)	6.50	0.011*
Present	12 (52.2)	4 (20.0)		

*TTM* trichotillomania, *IQR* interquartile range

^a^Mann–Whitney *U* test

^b^Fisher’s exact test

^c^Chi-square test

*p < 0.05

The mean duration of the research group’s TTM symptoms was found to be 2.76 ± 1.97 years. Of the TTM patients, 10 (43.4%) were previously diagnosed, and 13 (56.5%) were newly diagnosed with TTM (for details, see Supplementary Table S2). Of them, 14 (60.9%) had no comorbidities, and 9 (39.1%) had one or more comorbidities [5 patients with MDD, 2 patients with SAD, 2 patients with GAD, 2 patients with specific learning disorder (SLD), and 1 patient with PD (see Supplementary Table S3)]. It was noted that 5 patients (21.7%) were undergoing selective serotonin reuptake inhibitors’ (SSRIs) treatment (1 patient with 200 mg/day of sertraline, 3 patients with 100 mg/day of sertraline and 1 patient with 50 mg/day of sertraline).

All of the subscale scores on the RCADS-CV differed significantly between the two groups and were significantly higher in the research group (*p* < 0.001 for all, see Supplementary Table S4). The median (IQR) = 17 (14.83–20.16) and minimum–maximum values = 12–24 of the research group’s total score on the MGH-HPS scale were determined. The results of the BIS-Brief are shown in Table 2. Poor self-regulation, impulsive behavior, and the total score were statistically significantly higher in the TTM group than in the healthy controls.

**Table 2:** Comparison of the groups’ BIS-brief

Barratt impulsiveness scale-brief	TTM (*n* = 23) Median (IQR)	Control (*n* = 20) Median (IQR)	*U*	*p*
Poor self-regulation	8 (6.5–9.5)	7 (6–8)	136.0	0.019*
Impulsive behavior	11 (8.5–13.5)	8.5 (7–10)	118.5	0.006**
Total score	20 (17–23)	16 (13.6–18.4)	115.5	0.004**

*TTM* trichotillomania, *IQR* interquartile range, *U* Mann–Whitney *U* test

*p < 0.05

**p < 0.01

Table 3 shows both groups’ neurocognitive test scores while controlling the effect of total depression and anxiety scores using the ANCOVA test. This analysis found no statistically significant results for the go/no-go task. It found that the flanker effect was significantly lower in the TTM group for the Eriksen Flanker task. For the SSRT task, the TTM group’s percentage of effectively inhibited stop responses and percentage of correct go responses were significantly lower. For the BART, the number of adjusted pumps was significantly higher in the TTM group.

**Table 3:** Comparison of the neurocognitive test scores between the groups

	TTM (*n* = 23)		Control (*n* = 20)		*F*	*p*	Effect size
	Mean (SD)	Adjusted means (SE)	Mean (SD)	Adjusted means (SE)			
Eriksen flanker task							
Percentage of congruent stimulus accuracy	94.1 (13.8)	92.4 (3.0)	97.6 (6.7)	99.6 (3.3)	1.81	0.186	0.04
Percentage of incongruent stimulus accuracy	86.5 (14.2)	86.4 (3.4)	91.8 (10.2)	91.9 (3.8)	0.819	0.371	0.02
Flanker effect	59.2 (50.0)	45 (12.2)	81.7 (42.1)	98 (13.5)	6.00	0.019*	0.13
Stop-signal reaction-time task							
Stop-signal reaction time	352.7 (44.0)	361.6 (12.9)	329.6 (51.8)	319.4 (14.2)	3.42	0.072	0.08
Percentage of effectively inhibited stop responses	89.3 (8.0)	88.3 (1.7)	95.0 (2.8)	96.2 (1.8)	7.13	0.011*	0.15
Median correct reaction time for successful go trials	375.7 (30.2)	380.7 (7.1)	378.0 (21.6)	372.2 (7.9)	0.45	0.506	0.01
Percentage of correct go responses	81.6 (12.3)	80.0 (2.8)	89.2 (7.5)	91.0 (3.01)	4.98	0.031*	0.11
Go/no-go task							
Percentage of go accuracy	89.1 (20.8)	93.0 (4.4)	96.7 (9.5)	92.2 (4.9)	0.01	0.922	< 0.01
Correct reaction time for successful go trials	1307.1 (137,5)	1329.5 (44.3)	1224.1 (189.2)	1198.4 (49.0)	2.78	0.103	0.07
Percentage of no-go accuracy	82.1 (34.3)	94.7 (6.3)	97.1 (9.1)	82.5 (7.0)	0.18	0.283	0.03
Balloon analog risk task							
Number of adjusted pumps	23.4 (14.2)	24.5 (3.1)	14.6 (6.8)	13.4 (3.4)	4.11	0.049*	0.09
Maximum number of pumps	32.6 (19.0)	33.2 (4.2)	20.0 (9.5)	19.3 (4.6)	3.54	0.067	0.08
Number of exploded balloons	1.1 (0.9)	1.2 (0.2)	1.7 (0.8)	1.6 (0.3)	0.71	0.405	0.02
Total score	431.1 (268.4)	434.5 (57.5)	233.8 (109.9)	229.8 (63.5)	4.03	0.052	0.09

*TTM* trichotillomania, *SD* standard deviation, *SE* standard error

*p < 0.05

### Correlational analyses

The associations between the severity of TTM symptoms and impulsivity (as assessed by both self-report scale and behavioral tasks)/concomitant symptoms of anxiety and depression were examined. For all participants, the RCADS-CV-SAD score (*r* = 0.474, *p* < 0.05) and the RCADS-CV total internalizing score (*r* = 0.456, *p* < 0.05) were positively and moderately correlated with their scores on the MGH-HPS scale (Table 4). There were no significant relationships between the MGH-HPS scale scores and either their scores on the behavioral tasks or the TTM group’s BIS-Brief scores (Supplementary Table S5, S6). In all adolescents included in the study, moderately significant correlations were found between all the participants’ BIS-Brief and RCADS-CV scores (Supplementary Table S7).

**Table 4:** Correlation analysis between the TTM group’s RCADS-CV subscale scores, total score, and their MGH-HPS score

TTM (*n* = 23)	*R*
	MGH-HPS
RCADS-CV	
SAD	0.474*
SPD	0.058
GAD	0.367
PD	0.292
OCD	0.399
MDD	0.235
Total anxiety	0.289
Total internalizing	0.456*

*MGH-HPS* Massachusetts general hospital hair-pulling scale, *RCADS-CV* revised children’s anxiety and depression scale-child version, *SAD* social anxiety disorder, *SPD* separation anxiety disorder, *GAD* generalized anxiety disorder, *PD* panic disorder, *OCD* obsessive–compulsive disorder, *MDD* major depressive disorder

*p < 0.05, Spearman’s correlation test

### Regression analyses

Finally, the variables associated with the severity of TTM (MGH-HPS total score) in the previous correlation analyses were evaluated using a linear regression model. Simple linear regression analysis was performed to determine the effect of the each independent variable. The RCADS-SAD score and the RCADS-Internalizing score were included in the analysis, and an increase of one unit in the RCADS-SAD score increased the MGH-HPS score by 0.43 units (*p* = 0.038, beta = 0.435, adjusted *R*^2^ = 0.150).

## Discussion

This is the first study to demonstrate motor response inhibition deficits (using the SSRT task) in adolescents diagnosed with TTM even when controlling for total anxiety and depression scores, although similar findings have been reported in adult patients [8, 56, 57]. By creating a more homogeneous group in terms of age and gender (only adolescent girls) and by controlling for the use of medications such as methylphenidate, atomoxetine, and dopamine receptor antagonists, which have a corrective effect on motor inhibition deficits, this study may have produced a definitive result. Studies of the effect of gender on motor inhibition have found that brain regions closely associated with inhibitory motor control mature earlier in girls than in boys, with neuropsychological tests such as the SSRT task and go/no-go task showing more activity in these brain regions in girls, which means that girls are more capable of suppressing motor responses [29, 65, 66]. This means that demonstrating motor response inhibition deficits in girls with TTM is an important finding. In addition to these important findings, there is an inconsistency between the results of the SSRT task and the go/no-go task. These two tests are often used indiscriminately based on the assumption that they both measure similar inhibition processes. In fact, the go/no-go task measures action limitation, and the SSRT task measures action cancelation [67]. Moreover, it has been suggested that the inhibitory load is greater in the SSRT task, because the response that needs to be inhibited has already been initiated [75]. Behavioral studies support this distinction between neurocognitive mechanisms, since performance on one behavioral task can be impaired without a significant deficit in performance of the other [52, 60]. Interestingly, neurochemical studies have shown that while serotonin plays a more active role in the go/no-go task, norepinephrine is responsible for performance during the SSRT task [21]. Thus, it also seems possible that taking SSRIs could potentially lead to disparities in task outcomes, so studies in which the use of SSRIs is controlled will help us to better understand the all the components of motor response inhibition in TTM.

This study found that the risk-taking and disadvantageous decision-making tendency (BART) of the TTM group was higher than that of the healthy controls. Since no other studies have evaluated the decision-making processes of adolescents with TTM, this study presents the first data concerning this subject. While the myelination and synaptic pruning of brain structures, such as the dorsal striatum, the dorsomedial prefrontal cortex, and the frontoparietal cortex, persists into adulthood, the neurobiological structuring of ventral limbic regions such as the ventromedial prefrontal cortex is relatively faster during adolescence [64]. This structuring is associated with novelty seeking and a tendency to make risky decisions with potentially negative consequences. Although risk-taking behavior is accepted as part of the healthy developmental process, studies have shown that pathological risk-taking is closely associated with specific psychopathologies, such as alcohol/substance use disorders, pathological gambling, and suicide attempts [14, 30, 41]. TTM has been categorized in the taxonomy of OCRDs, but its shared characteristics with addiction-related disorders make positioning it in psychiatric classification systems an issue of controversy [36, 39]. Looking at TTM from an addiction perspective makes these features stand out: repetitive pulling despite negative consequences, loss of control over behavior, craving before pulling, feeling of excitement and pleasure during the pulling episode, dysregulation of the reward circuit, and significant reduction in pulling symptoms with the administration of naltrexone [15, 36, 77]. This point of view makes it seem that adolescents with TTM exhibit a heightened neurobiological predisposition toward disadvantageous decision-making compared to healthy peers, and that hair-pulling behavior can be considered a behavioral addiction. Other authors have conceptualized hair-pulling behavior as a “maladaptive method of emotion regulation.” Studies have shown that patients with TTM tend to experience disturbing emotions, such as boredom, disappointment, and dissatisfaction, and have difficulty regulating their emotions and high emotional reactivity [1, 20]. Behavioral studies have also shown that adolescents’ risk-taking behavior increases in stressful situations [24, 44]. Therefore, disadvantageous decision-making tendency may have been detected as a result of emotion regulation difficulties combined with a neurobiological predisposition in adolescents with TTM.

In this study, when the total anxiety and depression score was controlled, it was also found that the flanker effect was smaller in the TTM group, and although this difference was not statistically significant, the percentage of stimulus accuracy was lower. It was also observed that the reaction time for the congruent stimulus was prolonged in some patients in the TTM group. This may have caused the TTM group’s flanker effect to be smaller than that of the healthy control group. As far as we know, no other studies have evaluated interference control in adolescents with TTM. It may be the case that interference control remains more intact in TTM than other components of impulsivity (especially motor response inhibition and risky decision-making). To confirm these data, studies with large samples that control for the effects of confounding factors are needed. Given that impulsivity may be a central neurocognitive component in TTM, it may be that future studies’ use of behavioral tasks specific to different dimensions of impulsivity and brain regions in children and adolescents will contribute to a better understanding of the role of impulsivity in the pathophysiology of TTM.

This study found positive and moderately significant correlations between TTM severity and social anxiety/total anxiety and depression symptoms. Consistent with the results of this study, a few studies have reported that both anxiety and depressive symptoms are important predictors of symptom severity in patients with TTM [37, 53]. Simple linear regression analysis determined that social anxiety symptoms were the most important predictive variable for the severity of TTM. To the best of our knowledge, no studies have specifically investigated the effect of social anxiety symptoms on hair-pulling behaviors. Since TTM often affects visible parts of the body, such as the scalp, eyebrows and eyelashes, the idea of becoming less attractive leads to negative self-image, avoidance, and isolation [40]. One study found that 80% of patients with TTM had concerns about their body image, and that 20% met the diagnostic criteria for body dysmorphic disorder [69]. In addition, feelings of intense distress, shame, and disappointment caused by hair loss and thoughts of being judged by others may cause patients to avoid social environments and interpersonal relationships [28]. In a study that had adolescents complete a social acceptance scale after watching videos of three different patients—one with TTM, one with Tourette’s syndrome (TS), and one with no motor habit, the adolescents who exhibited TS or TTM were perceived as less socially acceptable by their peers [5]. Numerous studies have shown that negative social evaluation and peer rejection lead to the exacerbation of existing psychiatric symptoms or the development of psychopathology [16]. Patients also often try to hide their hair loss using wigs, elaborate hairstyles, exaggerated make-up, hats, or bandanas. This can be time-consuming and cause patients to miss out on recreational and social activities [71]. This finding makes it clear that high awareness and treatment of social anxiety symptoms in adolescents diagnosed with TTM may be an important step in the management of this disorder.

This study found no significant relationships between the level of impulsivity assessed by clinical and behavioral tasks and the severity of TTM. A possible explanation for its failure to detect a significant relationship between impulsivity and symptom severity, despite the high levels of self-reported and behavioral impulsivity in patients with TTM, is that impulsivity may be a predisposing factor that contributes to the initiation of the disorder, and that factors other than impulsivity may prolong or exacerbate symptom severity/disorder and thus disrupt functioning. Unlike this study, a limited number of studies have reported that impulsivity is an important factor in predicting the severity of TTM symptoms and functional impairment in adults [32, 54]. The small sample size of this study may have resulted in its inability to detect the effect of impulsivity on TTM severity. Another possible explanation is that the relationships between hair-pulling types and impulsivity may be different. Focused hair-pulling can be a planned activity intended to regulate dysfunctional emotions rather than an impulsive action, and it increases with age and is associated with greater symptom severity [26, 73]. The fact that this study did not include children and control for the type of hair-pulling behavior may have attenuated the effect of impulsivity on TTM severity. Finally, the self-report scale this study used to assess the severity of hair-pulling behavior only contains questions about the most recent week. This may have prevented a realistic assessment of the severity of hair-pulling behavior over time.

Limitations: This study has several positive features. Based on its data of the study, this is the first study to demonstrate motor response inhibition deficits and disadvantageous decision-making in adolescents diagnosed with TTM. For the first time, including only adolescent girls in the sample created a homogeneous cohort characterized by the convergence of age and gender where TTM is most common in children and adolescents. The potential impact of age and gender on impulse control was also controlled as much as possible. This study excluded patients diagnosed with ADHD, a psychiatric condition that is known to be closely associated with impulsivity, such as motor response inhibition and interference control, from its sample. At the same time, different facets of impulsivity, including motor response inhibition, the decision-making process, and interference control, were assessed together for the first time. Furthermore, controlling for the use of methylphenidate, atomoxetine, and dopamine receptor antagonists in the TTM cohort added to the robustness of this study’s findings. Despite this study’s remarkable results, it is important to recognize its limitations. Since this study has a cross-sectional design, it prevents the establishment of causal relationships between the variables studied. Due to its limited sample size, it is not possible to generalize the results of this study to all patients with TTM. Although efforts were made to construct a homogeneous cohort, patients diagnosed with MDD and anxiety disorders and concurrently treated with SSRIs, which have limited efficacy for treating TTM, were included.

### Supplementary Information

Below is the link to the electronic supplementary material.

Supplementary file1 (DOCX 44 KB)

## Data Availability

The data that support the findings of this study are available on request from the corresponding author. The data are not publicly available due to privacy or ethical restrictions.
