# Supplementary material for: Impulsivity in adolescent girls diagnosed with trichotillomania: an evaluation of clinical and neuropsychological characteristics
Source: Eur Child Adolesc Psychiatry. 2024 Jan 9;33(2):617–27. doi: 10.1007/s00787-023-02354-x (PMC10869386; doi:10.1007/s00787-023-02354-x)
Supplement: Supplementary file 1 — Supplementary file1 (DOCX 44 KB) [file 787_2023_2354_MOESM1_ESM.docx]

**Supplementary Material**

##### **Table S1.** Psychiatric disorders in relatives of groups

|  | TTM (n=23)  n (%) | | | |
| --- | --- | --- | --- | --- |
|  | First-degree | Second-degree | Third-degree | Fourth-degree |
| TTM | 2 (%8,7) | 3 (%13,0) |  | 1 (%4,3) |
| MDD | 1 (%4,3) | 2 (%8,7) | 1 (%4,3) |  |
| Anxiety Disorders | 2 (%8,7) |  |  |  |
| ADHD |  | 2 (%8,7) |  |  |
| Schizophrenia |  | 1 (%4,3) |  |  |
| ID |  |  |  | 1 (%4,3) |
| Alcohol Use Disorders | 1 (%4,3) |  |  |  |
| BAD |  |  | 1 (%4,3) |  |
|  | **Control (n=20)**  **n (%)** | | | |
|  | 1. derece | 2. derece | **3. derece** | **4. derece** |
| Anxiety Disorder | 2 (%10) |  | 1 (%5) |  |
| Schizophrenia |  |  |  | 1 (%5) |

TTM: Trichotillomania, MDD: Major Depressive Disorder, ADHD: Attention Deficit Hyperactivity Disorder, ID: Intellectual Disorder, BAD: Bipolar Affective Disorder

##### **Table S2.** Clinical characteristics of TTM group

| Clinical Variables | | n | % |
| --- | --- | --- | --- |
| Symptom duration (year) | <2 | 8 | 34.8 |
|  | 2-3 | 7 | 30.4 |
|  | >3 | 8 | 34.8 |
| Diagnosis | newly diagnosed | 13 | 87.5 |
|  | previously diagnosed | 10 | 8.9 |
| Pulling areas | scalp | 16 | 69.6 |
|  | eyebrow | 11 | 47.8 |
|  | eyelash | 10 | 43.5 |
|  | arm | 2 | 8.7 |
| Comorbid trichophagia | absent | 22 | 95.65 |
|  | present | 1 | 4.3 |

##### **Table S3.** Comorbid psychiatric diagnoses among TTM group

|  | TTM (n=23) |
| --- | --- |
| Comorbid Psychiatric Diagnoses | n (%) |
| Absent | 14 (60.9) |
| Present | 9 (39.1) |
| MDD | 5 (21.7) |
| SAD | 2 (8.7) |
| GAD | 2 (8.7) |
| SLD | 2 (8.7) |
| PD | 1 (4.3) |

MDD: Major Depressive Disorder, SAD: Social Anxiety Disorder, GAD: Generalized Anxiety Disorder, SLD: Specific Learning Disorder PD: Panic Disorder

##### **Table S4.** Comparison of Revised Children’s Anxiety and Depression Scale scores between groups

|  | TTM (n=23)  Mdn (IQR) | Control (n=20)  Mdn (IQR) | *U* | *p* | |
| --- | --- | --- | --- | --- | --- |
| RCADS-CV |  |  |  |  | |
| SAD | 61 (51-71) | 43 (40.5-45.5) | 46.0 | **< 0.001** | |
| SPD | 54 (48.5-59.5) | 44 (42-46) | 61.5 | **< 0.001** | |
| GAD | 61 (53.5-68.5) | 41 (38.5-43.5) | 32.0 | **< 0.001** | |
| PD | 68 (57-79) | 46 (41.5-50.5) | 42.5 | **< 0.001** | |
| OCD | 60 (50-70) | 40 (37-43) | 17.5 | **< 0.001** | |
| MDD | 68 (55-81) | 38 (35-41) | 22.5 | **< 0.001** | |
| Total Anxiety | 62 (54-70) | 40 (37.5-42.5) | 19.0 | | **< 0.001** |
| Total Internalizing | 68 (57.5-78.5) | 39 (36.5-41.5) | 11.0 | | **< 0.001** |

RCADS-CV: Revised Children’s Anxiety and Depression Scale Child Version, SAD: Social Anxiety Disorder, SPD: Separation Anxiety Disorder, GAD: Generalized Anxiety Disorder, PD: Panic Disorder, OCD: Obsessive-Compulsive Disorder, MDD: Major Depressive Disorder

Mann-Whitney *U* test

##### **Table S5.** Correlation analysis between behavioral tasks and MGH-HPS

| TTM (N=23) | r_s_ |
| --- | --- |
|  | MGH-HPS |
| Eriksen Flanker Test |  |
| Percentage of congruent stimulus accuracy | 0.188 |
| Percentage of incongruent stimulus accuracy | -0.144 |
| Flanker effect | 0.160 |
| Stop Signal Reaction Time Task |  |
| Stop Signal Reaction Time | -0.184 |
| Percentage of effectively inhibited “stop” responses | 0.290 |
| Correct reaction time for successful “go” trials | -0.382 |
| Percentage of correct “go” responses | 0.336 |
| Go/No Go Task |  |
| Percentage of go accuracy | -0.064 |
| Correct reaction time for successful “go” trials | -0.058 |
| Percentage of no-go accuracy | -0.071 |
| Balloon Analogue Risk Task |  |
| Number of adjusted pumps | 0.162 |
| Maximum number of pumps | 0.231 |
| Number of exploded balloons | 0.059 |
| Total points earned | 0.213 |

**p*<0.05, *p*<0.01, *p*<0.001

MGH-HPS: Massachusetts General Hospital Hairpulling Scale,

*Spearman correlation test*

##### **Table S6.** Correlation analysis between BIS- Brief and MGH-HPS

| TTM (N=23) | r_s_ |
| --- | --- |
|  | MGH-HPS |
| Barratt İmpulsiveness Scale-Brief |  |
| Poor Self-Regulatıon | 0.252 |
| Impulsive Behavior | 0.342 |
| Total Point | 0.339 |

**p*<0.05, *p*<0.01, *p*<0.001

BIS- Brief: Barratt Impulsiveness Scale- Brief, MGH-HPS: Massachusetts General Hospital Hairpulling Scale *Spearman correlation test*

*Spearman correlation test*

##### **Table S7.** Correlation Analysis Between RCADS-CV and BIS-Brief subscale and total scores in all participants

| **Participants (n=43)** | r | | |
| --- | --- | --- | --- |
|  | **BIS-Brief** | | |
|  | Poor self-regulation | Impulsive behaviour | Total score |
| **RCADS-CV** |  |  |  |
| SAD | **0.495**** | **0.562**** | **0.570**** |
| SPD | **0.431**** | **0.425**** | **0.461**** |
| GAD | **0.322*** | **0.443**** | **0.417**** |
| PD | 0.287 | **0.415**** | **0.392**** |
| OCD | **0.435**** | **0.505**** | **0.522**** |
| MDD | **0.498**** | **0.651**** | **0.640**** |
| Total Anxiety | **0.430**** | **0.430**** | **0.535**** |
| Total Internalizing | **0.440**** | **0.559**** | **0.551**** |

BIS- Brief: Barratt Impulsiveness Scale- Brief, RCADS-CV: Revised Children’s Anxiety and Depression Scale Child Version, SAD: Social Anxiety Disorder, SPD: Separation Anxiety Disorder, GAD: Generalized Anxiety Disorder, PD: Panic Disorder, OCD: Obsessive-Compulsive Disorder, MDD: Major Depressive Disorder

*<0.05

**<0.01

***<0.001

*Spearman correlation test*

##### **Table S8.** Correlation analysis between behavioral tasks and RCADS-CV scores in all participants

| Participants (n=43) | r | | | | | | | | | | | | | | |
| --- | --- | --- | --- | --- | --- | --- | --- | --- | --- | --- | --- | --- | --- | --- | --- |
|  | **RCADS-CV** | | | | | | | | | | | | | | |
|  | **SAD** | **SPD** | | **GAD** | | | **PD** | **OCD** | | **MDD** | | **Total Anxiety** | | | **Total Internalizing** |
| Eriksen Flanker Test |  | |  | |  |  | | |  | |  | |  |  | |
| Percentage of congruent stimulus accuracy | -0.144 | | 0.096 | | -0.067 | -0.117 | | | -0.081 | | -0.259 | | -0.056 | -0.093 | |
| Percentage of incongruent stimulus accuracy | -0.237 | | -0.161 | | -0.154 | -0.139 | | | -0.293 | | -0.198 | | -0.224 | -0.228 | |
| Flanker Effect | -0.016 | | 0.003 | | 0.064 | 0.013 | | | 0.109 | | -0.066 | | -0.004 | -0.025 | |
| Stop Signal Reaction Time Task |  | |  | |  |  | | |  | |  | |  |  | |
| Stop Signal Reaction Time | 0.134 | | **-0.358*** | | 0.139 | 0,064 | | | 0.038 | | 0.285 | | 0.089 | 0.129 | |
| Percentage of effectively inhibited “stop” responses | -0.236 | | **-0.326*** | | -0.158 | -0.100 | | | -0.083 | | -0.273 | | -0.174 | -0.201 | |
| Correct reaction time for successful “go” trials | -0.091 | | 0.163 | | -0.059 | -0.115 | | | -0.255 | | -0.023 | | -0.117 | -0.114 | |
| Percentage of correct “go” responses | -0.164 | | -0.296 | | -0.134 | -0.126 | | | -0.134 | | **-0.330*** | | -0.156 | -0.188 | |
| Go/No Go Task |  | |  | |  |  | | |  | |  | |  |  | |
| Percentage of go accuracy | -0.024 | | -0.188 | | 0.006 | -0.009 | | | -0.144 | | -0.190 | | -0.066 | -0.097 | |
| Correct reaction time for successful “go” trials | -0.035 | | 0.152 | | 0.039 | -0.025 | | | -0.062 | | 0.123 | | -0.027 | 0.021 | |
| Percentage of no-go accuracy | **-0.335*** | | -0.294 | | -0.272 | -0.250 | | | **-0.354*** | | **-0.314*** | | **-0.355*** | **-0.338*** | |
| Balloon Analogue Risk Task |  | |  | |  |  | | |  | |  | |  |  | |
| Number of adjusted pumps | 0.232 | | 0.144 | | 0,147 | 0.141 | | | **0.301*** | | 0.026 | | 0.288 | 0.229 | |
| Maximum number of pumps | 0.296 | | 0.187 | | 0,186 | 0.195 | | | **0.326*** | | 0.068 | | **0.337*** | 0.278 | |
| Number of exploded balloons | -0.263 | | -0.139 | | -0,267 | **-0.330*** | | | -0.185 | | **-0.310*** | | -0.200 | -0.240 | |
| Total points earned | **0.335*** | | 0.197 | | 0,222 | 0.257 | | | **0.384*** | | 0.134 | | **0.385*** | **0.327*** | |

RCADS-CV: Revised Children’s Anxiety and Depression Scale Child Version, SAD: Social Anxiety Disorder, SPD: Separation Anxiety Disorder, GAD: Generalized Anxiety Disorder, PD: Panic Disorder, OCD: Obsessive-Compulsive Disorder, MDD: Major Depressive Disorder

*<0.05

**<0.01

***<0.001

*Spearmen correlation test*

##### **Table S9.** Correlation analysis between behavioral tasks and BIS- Brief scores in all participants

| Participants (n=43) Toplam | r | | | |
| --- | --- | --- | --- | --- |
|  | **Barratt Impulsiveness Scale- Brief** | | | |
|  | Poor self-regulation | Impulsive behavior | | Total score |
| Eriksen Flanker Test |  | |  |  |
| Percentage of congruent stimulus accuracy | **-0.301*** | | -0.225 | **-0.303*** |
| Percentage of incongruent stimulus accuracy | **-0.355*** | | -0.288 | **-0.371*** |
| Flanker effect | 0.037 | | -0.095 | -0.057 |
| Stop Signal Reaction Time Task |  | |  |  |
| Stop Signal Reaction Time | 0.286 | | 0.196 | 0.254 |
| Percentage of effectively inhibited “stop” responses | **-0.398**** | | **-0.414**** | **-0.458**** |
| Correct reaction time for successful “go” trials | 0.247 | | -0.091 | 0.071 |
| Percentage of correct “go” responses | -0.243 | | -0.170 | -0.240 |
| Go/No Go Task |  | |  |  |
| Percentage of go accuracy | -0.208 | | -0.221 | -0.240 |
| Correct reaction time for successful “go” trials | 0.107 | | 0.028 | 0.071 |
| Percentage of no-go accuracy | -0.243 | | **-0.369*** | **-0.356*** |
| Balloon Analogue Risk Task |  | |  |  |
| Number of adjusted pumps | 0.037 | | 0.120 | 0.086 |
| Maximum number of pumps | 0.066 | | 0.155 | 0.122 |
| Number of exploded balloons | -0.065 | | -0.011 | -0.044 |
| Total points earned | 0.066 | | 0.130 | 0.109 |

BIS- Brief: Barratt Impulsiveness Scale- Brief

*<0.05

**<0.01

***<0.001

*Spearmen correlation test*
